# Formononetin Defeats Multidrug-Resistant Cancers by Induction of Oxidative Stress and Suppression of P-Glycoprotein

**DOI:** 10.3390/ijms25158471

**Published:** 2024-08-02

**Authors:** Ying-Tzu Chang, I-Ting Wu, Ming-Jyh Sheu, Yu-Hsuan Lan, Chin-Chuan Hung

**Affiliations:** 1 Department of Pharmacy, China Medical University, No. 100, Section 1, Jingmao Road, Beitun District, Taichung 406040, Taiwan; changyt@365.cmu.edu.tw (Y.-T.C.); u105015202@cmu.edu.tw (I.-T.W.); hsumj@mail.cmu.edu.tw (M.-J.S.); lanyh@mail.cmu.edu.tw (Y.-H.L.); 2 Department of Pharmacy, China Medical University Hospital, Taichung 404327, Taiwan; 3 Department of Healthcare Administration, Asia University, Taichung 41354, Taiwan

**Keywords:** formononetin, multidrug resistance cancer, P-glycoprotein, oxidative stress

## Abstract

Multidrug resistance (MDR) remains the most difficult problem facing conventional chemotherapy for cancers. *Astragalus membranaceus* is a historically traditional Chinese medicine. One of its bioactive components, formononetin, exhibits antitumor effects on various cancers. However, the effects of formononetin on MDR cancers have not been evaluated. Therefore, we investigated the defense’s effects of formononetin on MDR. We used rhodamine 123 and doxorubicin efflux assays to analyze the inhibition kinetics of P-glycoprotein (P-gp) mediated-efflux. Cell viability was detected by sulforhodamine B assay, and the synergistic effects of formononetin combined with chemotherapeutic agents were further calculated using CompuSyn software. Molecular docking was performed with iGEMDOCK. We discovered that formononetin considerably induced oxidative stress and the disruption of mitochondrial membrane potential in MDR cancer cells. Furthermore, formononetin inhibits the P-gp efflux function by ATPase stimulation and the uncompetitive inhibition of P-gp-mediated effluxes of rhodamine 123 and doxorubicin. The molecular docking model indicates that formononetin may bind to P-gp by strong hydrogen bonds at Arginine (Arg) 489 and Glutamine (Gln) 912. Formononetin exhibits significant synergistic effects with vincristine and doxorubicin toward MDR cancer cells, and it synergistically suppressed tumor growth in vivo with paclitaxel. These results suggest that formononetin should be seen as a potential candidate for the adjuvant therapy of MDR cancers.

## 1. Introduction

Cancer multidrug resistance (MDR) hinders the efficacy of conventional cancer chemotherapy. The excessive proliferation of drug efflux channels can lead to the occurrence of MDR. Among them, the overexpression of ATP-binding cassette transporters, including P-glycoprotein (P-gp), MDR-related protein 1 (MRP1), and breast cancer resistance protein (BCRP), is most relevant to resistance to chemotherapy drugs [1]. P-gp is an active transport protein that exists in intestinal epithelial cells (enterocytes). When drugs are administered orally, the drug molecules pass through the intestinal epithelial cells and enter the blood. If, unfortunately, they encounter P-gp, it will destroy the drug molecules. This mechanism could prevent drug molecules from entering systemic circulation, effectively reducing drug bioavailability.

P-gp is encoded by the *ABCB1* gene, which consists of two homologous halves. It is an apical transporter protein that is abundantly present in the intestine, kidney, blood–brain barrier and placental cavity. It protects cells by removing xenobiotics from cells [2,3]. Various chemotherapeutic drugs, such as paclitaxel, doxorubicin, or vincristine, could induce P-gp expression, leading to cancer recurrence and ultimately relapse or death [4,5]. Recent studies have shown that P-gp inactivation could weaken the defenses of MDR cancer cells to conventional chemotherapeutic drugs. Therefore, the inhibition of P-gp is considered a promising solution that could play a critical role in cancer development and treatment outcomes [6,7,8,9].

Four generations of P-gp inhibitors have been developed to date. Nevertheless, the past three generations of P-gp inhibitors failed clinically due to non-selective inhibition, unpredictable drug interactions, and systemic toxicity. Therefore, taking advantage of the potential low toxicity of natural products to expand the safety-centered new generation of P-gp inhibitors for cancer treatment has been the focus of development in recent years. [10].

Formononetin (FMN) is a natural isoflavone isolated from *Astragalus membranaceus* that has biologically active antiviral, antiangiogenic, immunomodulatory, and anticancer properties [11,12,13]. In cancer-related studies, formononetin exhibits cytotoxicity on various cancers. Formononetin induced the apoptosis and suppressed phosphorylation of AKT in the cervical cancer HeLa cell line and inhibited cell proliferation in human ovarian and breast cancers by suppressing the PI3K/AKT and ERK1/2 pathways [14,15]. Although several natural compounds are reported to exert P-gp inhibitory effects [16,17], the roles of formononetin in the P-gp transported role and MDR cancer inversion effect remain unpredictable. Therefore, we aimed to examine whether formononetin also exerts an MDR inversion effect and the associated molecular mechanisms.

## 2. Results

### 2.1. Modulation of P-gp Functions by Formononetin in ABCB1 Overexpressing Cells

**Figure 1:**
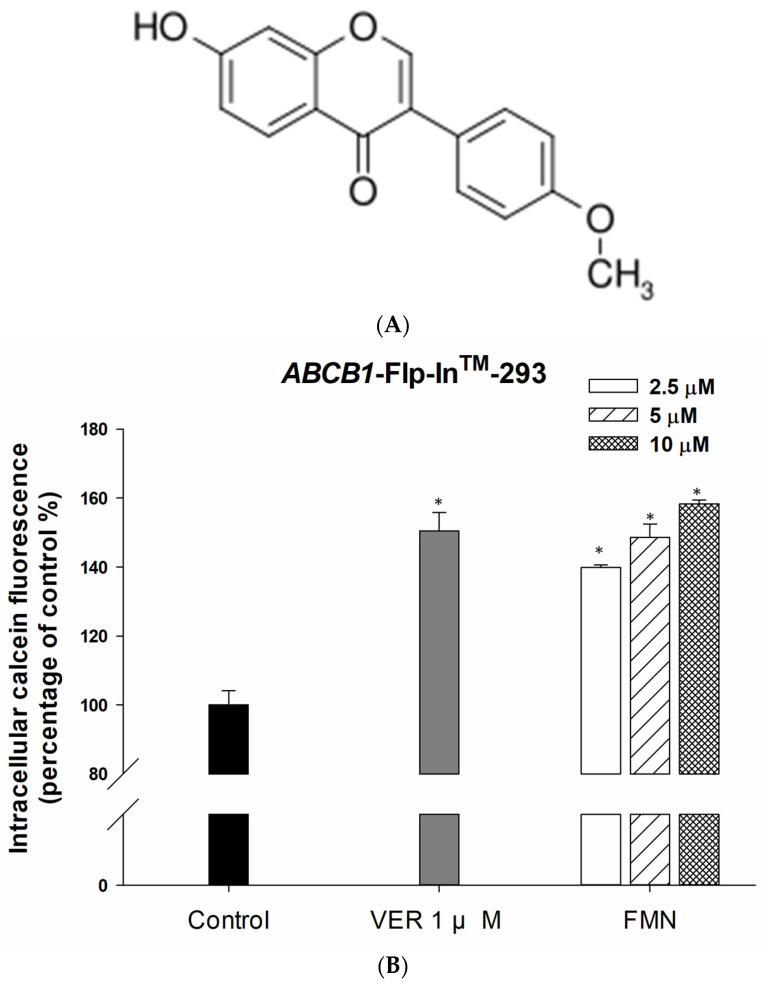
Inhibitory effect of formononetin on P-gp efflux of *ABCB1*/Flp-In^TM^-293 cells. (**A**) Chemical structure of formononetin. (**B**) P-gp overexpressing *ABCB1*/Flp-In^TM^-293 cells were incubated with serial-dose formononetin or verapamil, a positive control, for 30 min. The intracellular calcein fluorescence was determined to indicate the inhibition of P-gp. Each data are expressed as the mean ± standard error of at least two experiments, each performed in triplicate. Verapamil has used as positive control of P-gp inhibitor. The abbreviations of verapamil and formononetin are VER and FMN, respectively. * denotes *p* < 0.05 as compared to control group.

The chemical structure of formononetin is shown in Figure 1A. To confirm concentrations of formononetin for the following experiments, the cytotoxicity was tested by SRB assay. Treatment of 100 µg/mL formononetin for 72 h showed higher than 80% cell viability in all cell lines. Next, we verified the effect of formononetin on P-gp efflux ability. The accumulation of intracellular calcein fluorescence can be used to present as a P-gp efflux function, since the hydrophobic P-gp substrate, calcein-AM, converted to fluorescent calcein by intracellular esterase. Compared with untreated control cells, the intracellular calcein fluorescence was significantly increased by formononetin at a concentration between 0.62 and 2.5 µg/mL, as well as a standard P-gp inhibitor, verapamil (Figure 1B).

### 2.2. Formononetin Inhibits P-gp Efflux Function by Inducing ATPase Activity

**Figure 2:**
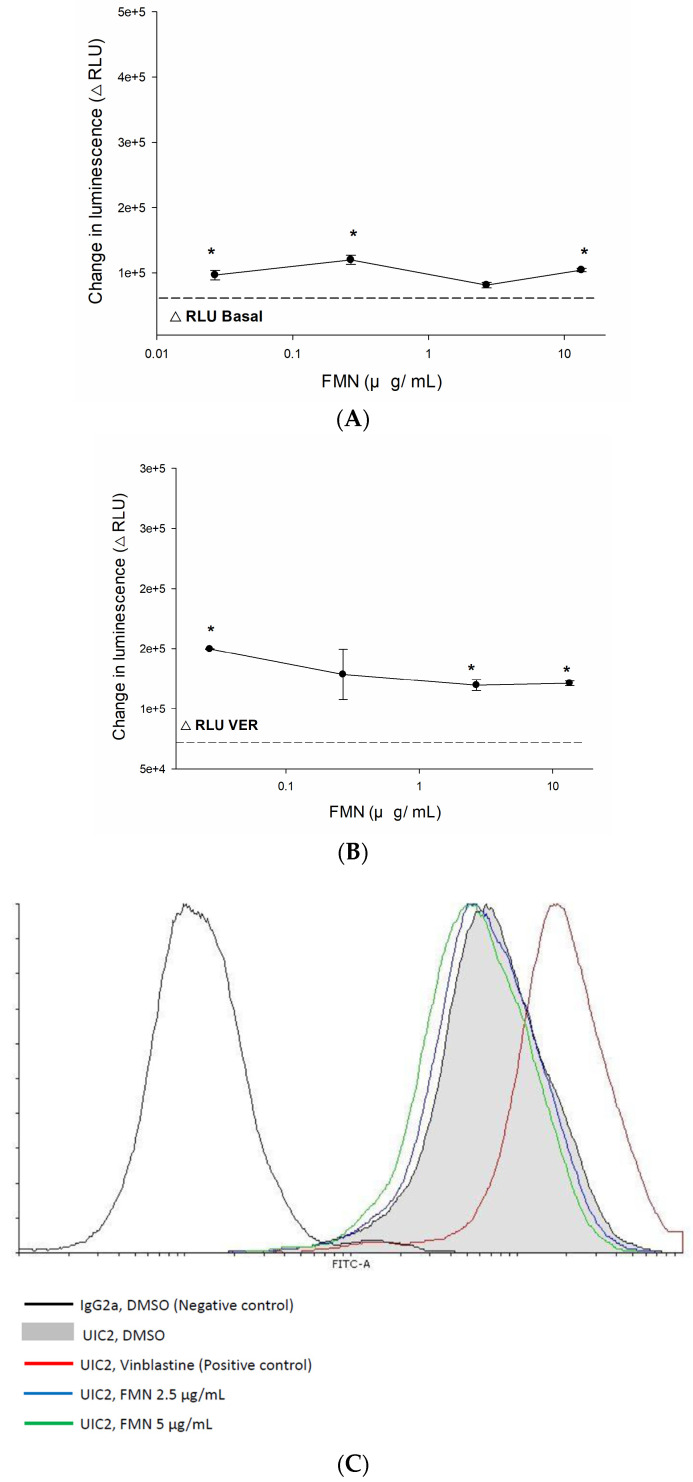
(**A**,**B**) The effects of FMN on P-gp ATPase activity was detected and data were analyzed as RLUs. After P-gp membranes were incubated with or without FMN, the unmetabolized ATP transformed into a luminescence. FMN could significantly stimulate basal and verapamil-stimulated P-gp ATPase activity. Verapamil (200 µM) was used as positive control. (**A**,**B**) Data were presented as the difference between Na3VO_4_-treated samples. Each data are expressed as the mean ± standard error of at least two experiments, each performed in triplicate. * denotes *p* < 0.05 as compared to control group. (**C**) P-gp substrates were identified by MDR1 shift assay, UIC2 fluorescence was increased during the binding of substrate on P-gp. Vinblastine (22.5 µM), a P-gp standard substrate, was used as positive control. The abbreviations of verapamil and formononetin are VER and FMN, respectively.

P-gp functions as an ATP-dependent drug efflux protein that is required to be activated by phosphates (the ATP metabolite hydrolyzed by ATPase). However, regardless of the increases or decreases in ATPase levels, P-gp function eventually fails due to the deficiency of phosphates. The Pgp-Glo assay was utilized to detect the consequence of formononetin on P-gp ATPase and presented as the difference between Na_3_VO_4_-treated samples and test compounds-treated samples, where the luminescence reflects the remaining unmetabolized ATP. Compare to no treatment control, formononetin significantly stimulated basal and verapamil-stimulated P-gp ATPase activity (Figure 2A,B), suggesting that formononetin may bind to different sites on ATPase with verapamil.

The results of MDR1 shift assay (Figure 2C) showed that the fluorescence of UIC2 increases in the vinblastine treatment group, whereas 2.5 µg/mL and 5 µg/mL formononetin did not significantly shift the peak to the right as compared to the control (DMSO only). It indicated that formononetin is not a substrate of P-gp.

### 2.3. Inhibition Kinetics of the P-gp Substrate Rhodamine 123 and Doxorubicin

**Figure 3:**
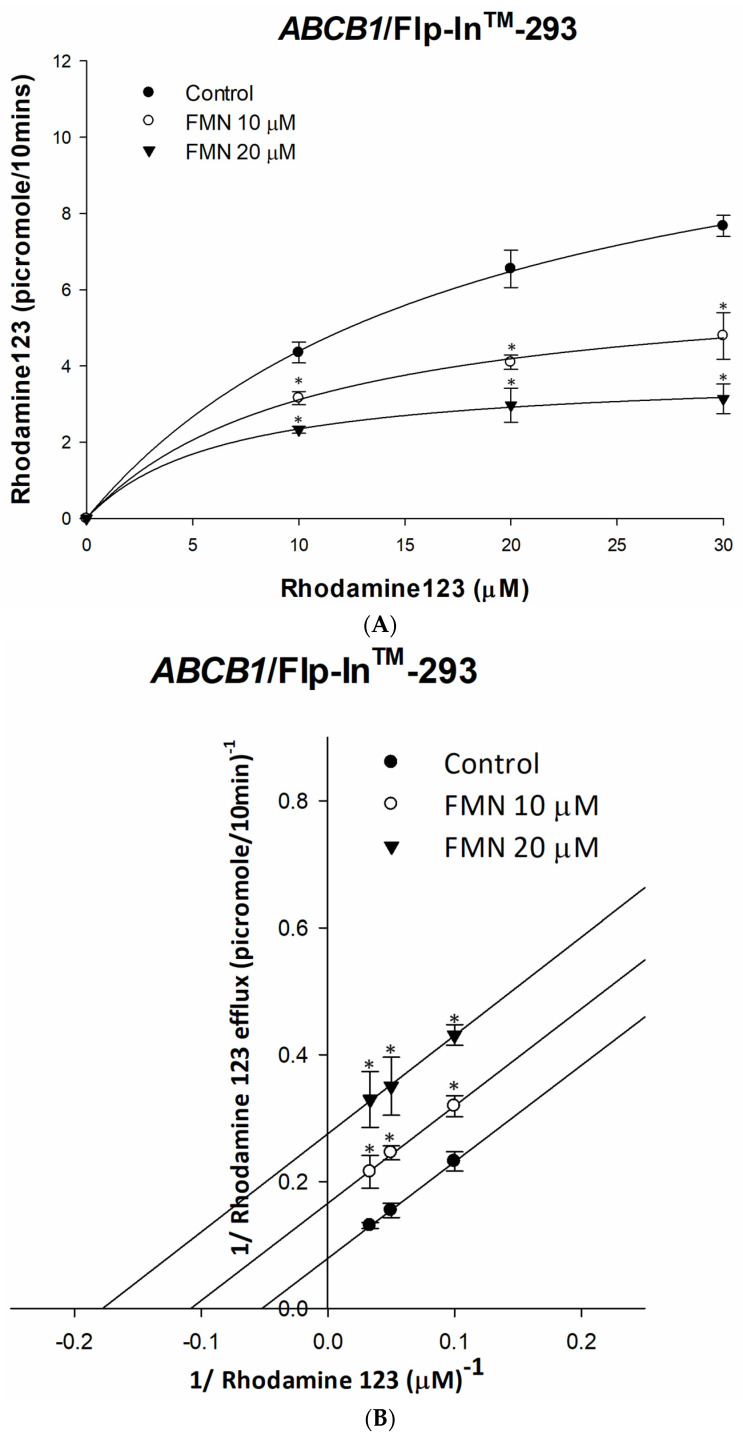

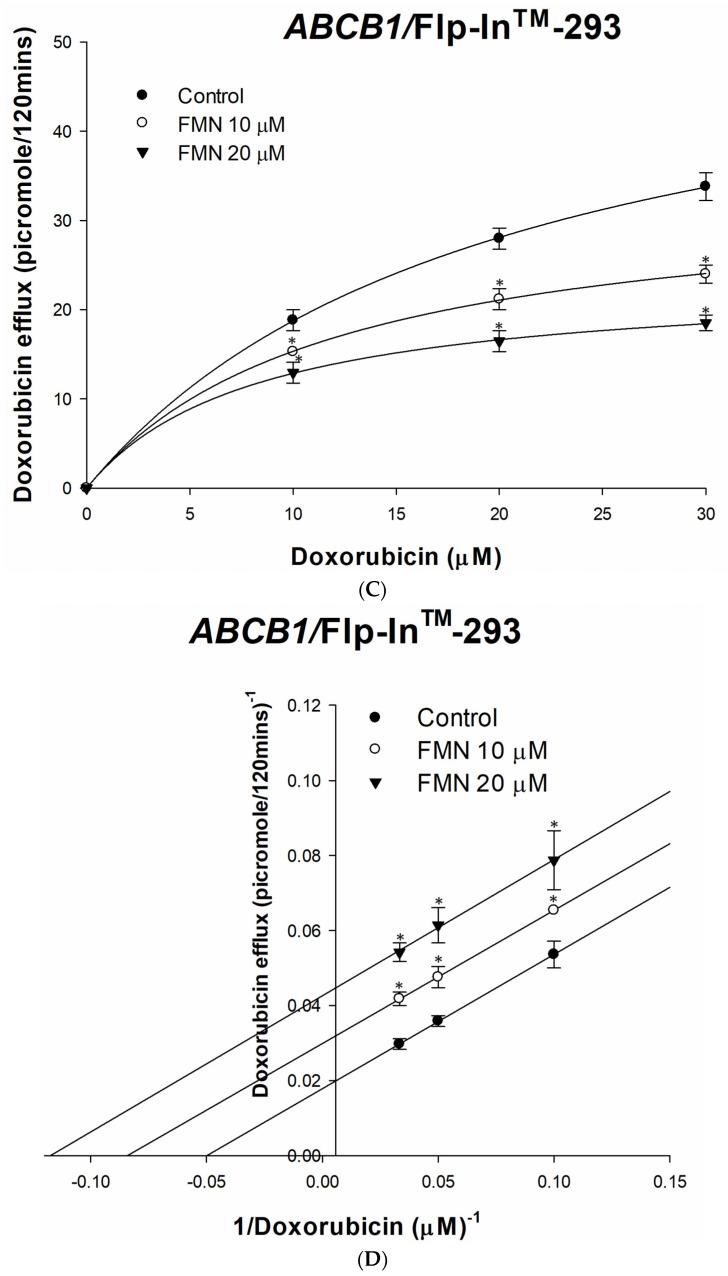
The kinetic interactions of formononetin on rhodamine 123 or doxorubicin. Michaelis–Menten kinetics of P-gp efflux were determined by the extracellular fluorescence of rhodamine 123 (**A**) or doxorubicin (**C**). Lineweaver–Burk plot analysis of formononetin inhibitory mechanism on rhodamine 123 and doxorubicin efflux is shown in (**B**,**D**), respectively. Each datum is expressed as the mean ± standard error of at least two experiments, each performed in triplicate. * denotes *p* < 0.05 as compared to nontreatment control.

The effects of formononetin on rhodamine 123 or doxorubicin efflux are shown in Figure 3A,C. The extracellular fluorescence of rhodamine 123 or doxorubicin was significantly decreased with formononetin treatment in a dose-dependent manner, and saturation at a higher concentration of rhodamine 123 or doxorubicin demonstrated that these data followed Michaelis–Menten kinetics. The inhibitory mechanism was further analyzed with nonlinear regression, the maximum rate (Vmax) and affinity (km) of rhodamine 123 and doxorubicin efflux were decreased by increasing the drug concentration of formononetin, and the half-maximal inhibitory concentration was 2.42 ± 0.1 µg/mL and 2.49 ± 0.04 µg/mL (Table 1). The Lineweaver–Burk plot (Figure 3B,D) revealed that the increases of x- and y-intercepts were accompanied by rising of formononetin treatment concentration. Taken together, these results suggested that formononetin uncompetitively inhibited the efflux of rhodamine 123 and doxorubicin by human P-gp.

### 2.4. The Synergetic Effects of Formononetin with Chemotherapeutic Agents on MDR Cancer Cell Line

**Figure 4:**
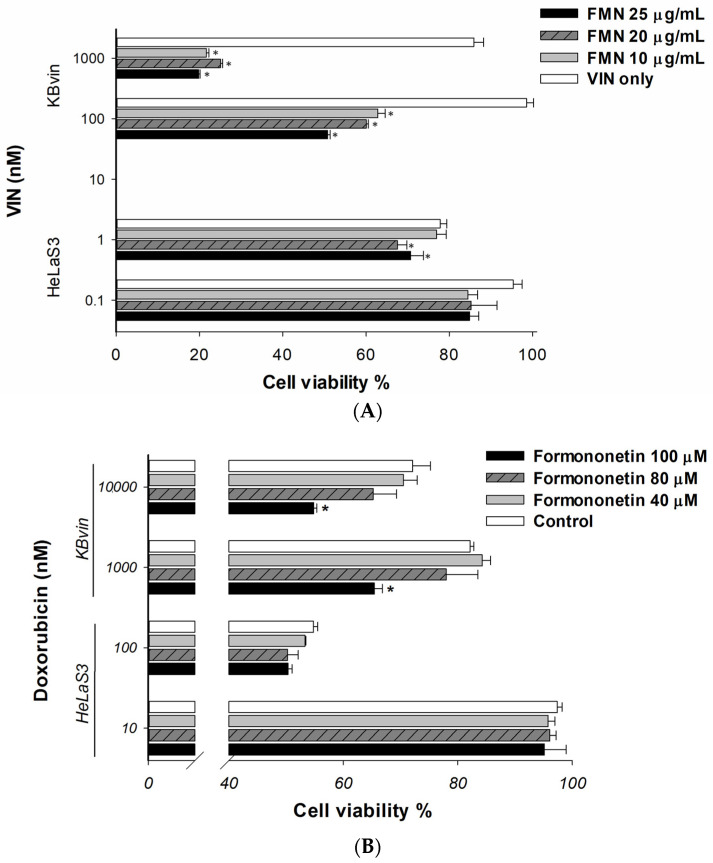

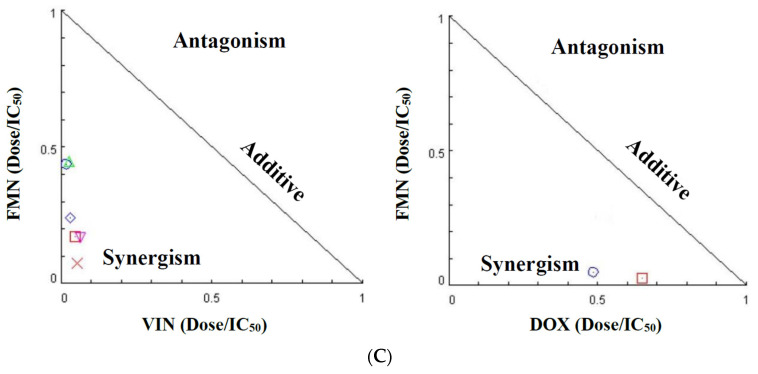
Effects of formononetin combined with chemotherapeutic agents on cell viabilities of drug-sensitive HeLaS3 cells and MDR KBvin cells. (**A**,**B**) The combination effects of formononetin and vincristine or doxorubicin on cell viability by SRB assay in cervical cancer HeLaS3 cells and MDR KBvin cells. Cells were pretreated with drugs alone or compound drug for 72 h. (**C**) Normalized isobologram for non-constant ratio combination of formononetin and chemotherapeutic drugs. The co-treatment of vincristine or doxorubicin with formononetin for 72 h at different combined concentrations. The actual drug dose was normalized with its corresponding IC_50_ and used to determine the synergetic effect of the co-treatments in KBvin cells. The line on the isobologram denotes the half effect from each drug. Antagonism, additive, or synergism effects were indicated above, on, or below the line, respectively. ⊡ formononetin 25 µg/mL + vincristine 1000 nM/doxorubicin 10,000 nM ⊙ formononetin 25 µg/mL + vincristine 100 nM/doxorubicin 1000 nM; ▽ formononetin 20 µg/mL + vincristine 1000 nM; △ formononetin 20 µg/mL + vincristine 100 nM; ⟐ formononetin 20 µg/mL + vincristine 1000 nM; ╳ formononetin 20 µg/mL + vincristine 1000 nM. Data presented as mean ± SE of at least two experiments, each in duplicate. * indicates *p* value < 0.05 compared with doxorubicin only or vincristine-only group. The abbreviations of vincristine, doxorubicin, and formononetin are VIN, DOX, and FMN, respectively.

**Figure 5:**
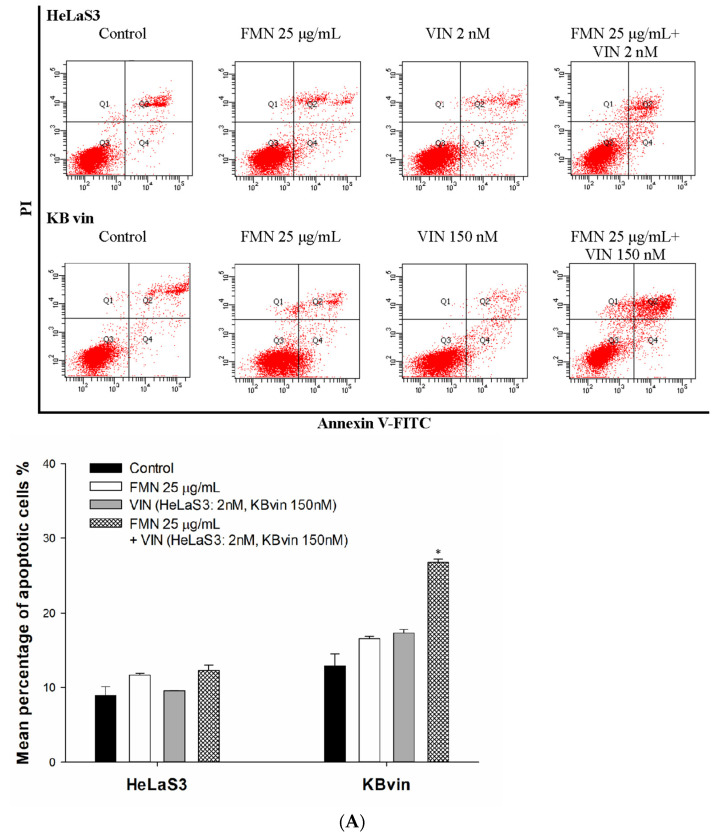

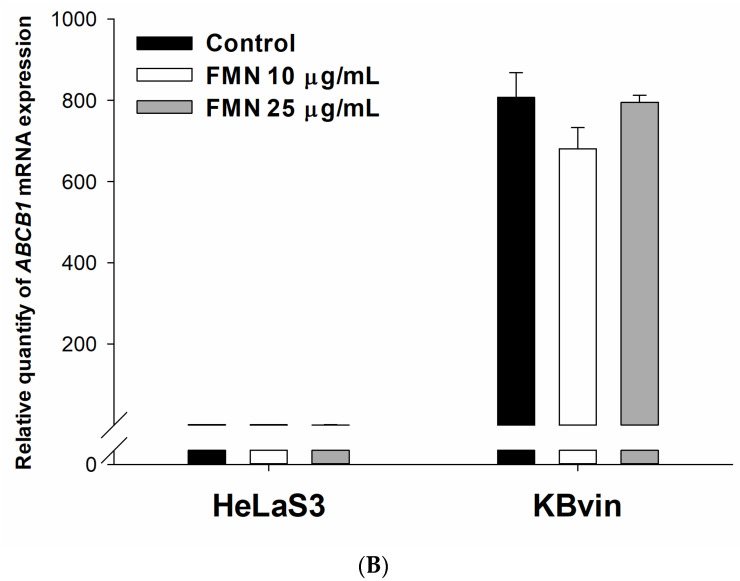
Mechanism of formononetin MDR reversal ability on cancer cells. (**A**) The effects of formononetin on vincristine-induced cytotoxicity was assessed by apoptosis assay. Apoptotic cells were stained with 5 µL of Annexin V–FITC and propidium iodide (PI) and analyzed by flow cytometry. (**B**) *ABCB1* mRNA expression was determined by real-time RT PCR. Cells were pretreated with formononetin 10 µg/mL or 25 µg/mL in HeLaS3 and KBvin for 72 h. Each datum is expressed as the mean ± standard error of at least two experiments, each performed in triplicate. * denotes *p* < 0.05 as compared to control group. The abbreviations of vincristine and formononetin are VIN and FMN, respectively.

The MDR reversal ability of formononetin was evaluated by cell viability assay. As shown in Figure 4A,B, co-treatment with formononetin synergistically reduced the viability of MDR KBvin cells in a dose-dependent manner as compared to vincristine or doxorubicin alone but this was not represented in HeLaS3 cells. Furthermore, the combination effects were confirmed using the CI values analysis. A normalized isobologram of compound–drug combination treatments falls within the area of synergism (Figure 4C), and CI values ranged from 0.1 to 0.7, suggesting the synergism effect of the combination treatments (Table 2). After that, we investigated whether synergistic lethality was caused by induced apoptosis. The number of vincristine-induced apoptotic cells was significantly increased by formononetin in MDR cell lines (Figure 5A). Furthermore, we explored the affected of formononetin on MDR cancer cell lines overexpressing P-gp. The results of real-time RT PCR demonstrated that 10 µg/mL or 25 µg/mL formononetin 72 h treatment did not influence the *ABCB1* mRNA expression in parental HeLaS3 cell nor MDR KBvin cells as compared to the control (Figure 5B). The abbreviations of formononetin is FMN.

### 2.5. The Docking Model of Formononetin on P-gp

**Figure 6:**
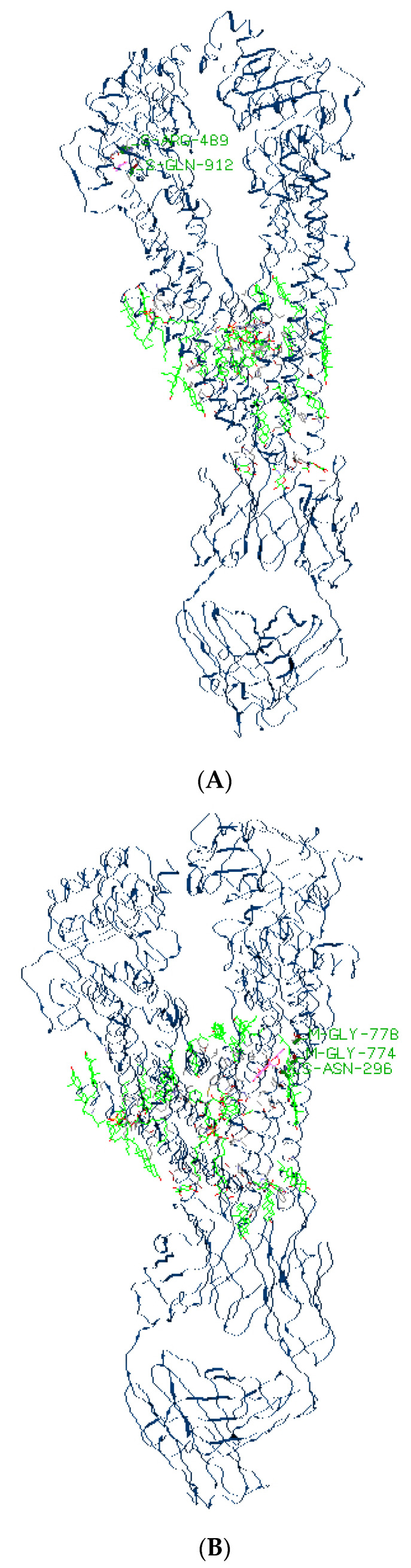

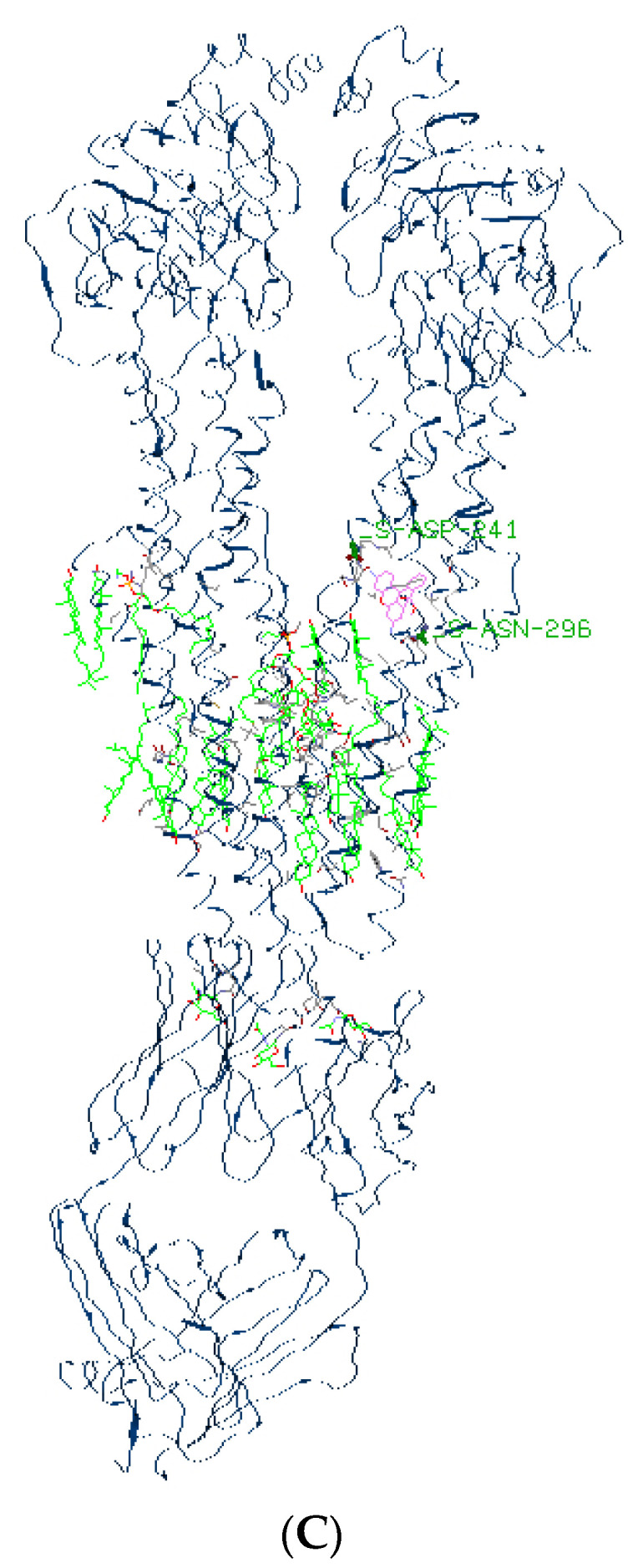
The docking results showed the superimposition of docked poses of compounds in the P-gp-binding pocket of the 3D structure of formononetin, rhodamine 123, and doxorubicin on P-gp: (**A**) formononetin, (**B**) rhodamine 123, (**C**) doxorubicin.

Molecular docking was carried out to simulate the interactions between that ligand and the structure of human P-glycoprotein. We used the docking program to supposedly dock the structures of the ligand-binding domain of P-gp (PDB entry 6QEX) on formononetin, doxorubicin, and rhodamine 123. Figure 6A–C indicated that the formononetin (−113.1 kcal/mol) exhibited alike binding affinity to human P-gp compared to doxorubicin (−138 kcal/mol) and rhodamine 123 (−103.3 kcal/mol). The docked poses of formononetin showed strong hydrogen bonds with Arg 489 and Gln 912 in the binding site of P-gp, while doxorubicin bonded with Asparagine (Asn) 296, Glycine (Gly) 774 and Gly 778, and rhodamine 123 bonded with Asp 241 and Asn 296.

### 2.6. Formononetin Induced ROS Production and Mitochondria Membrane Potential Changes

**Figure 7:**
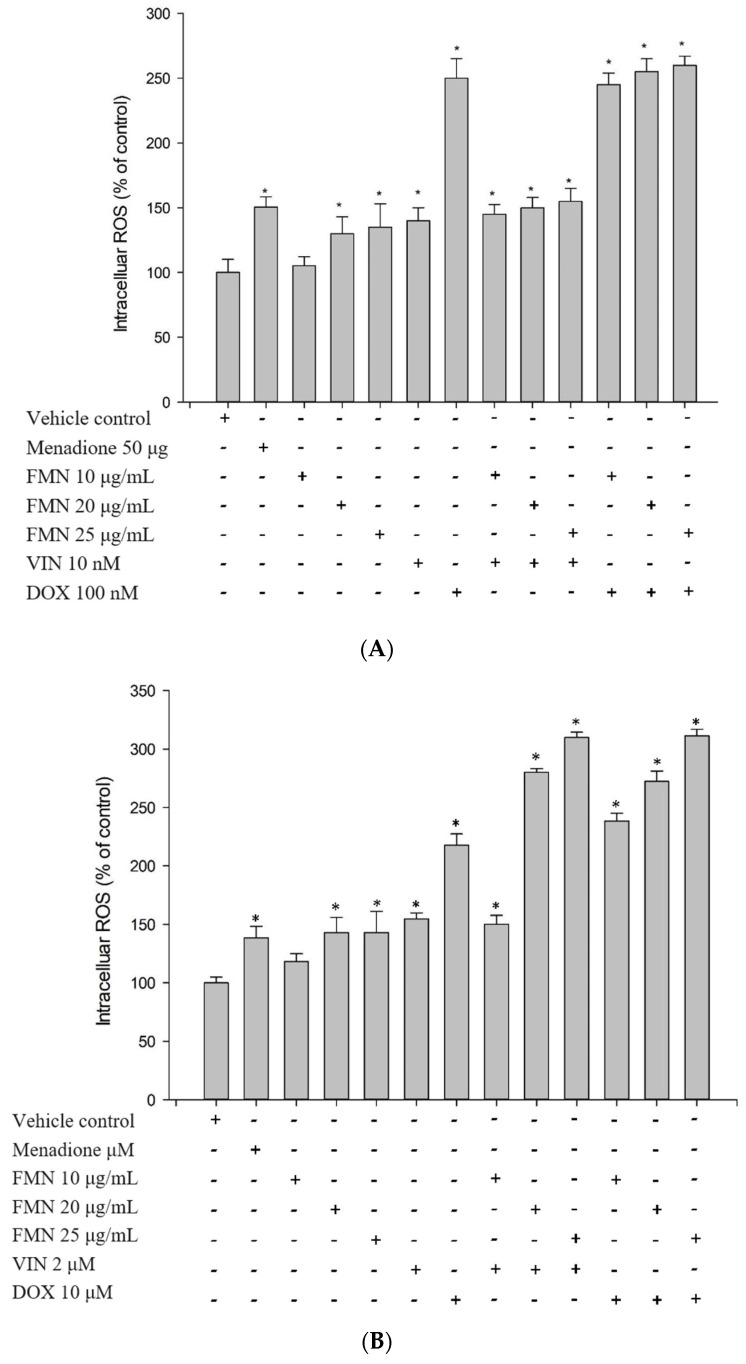

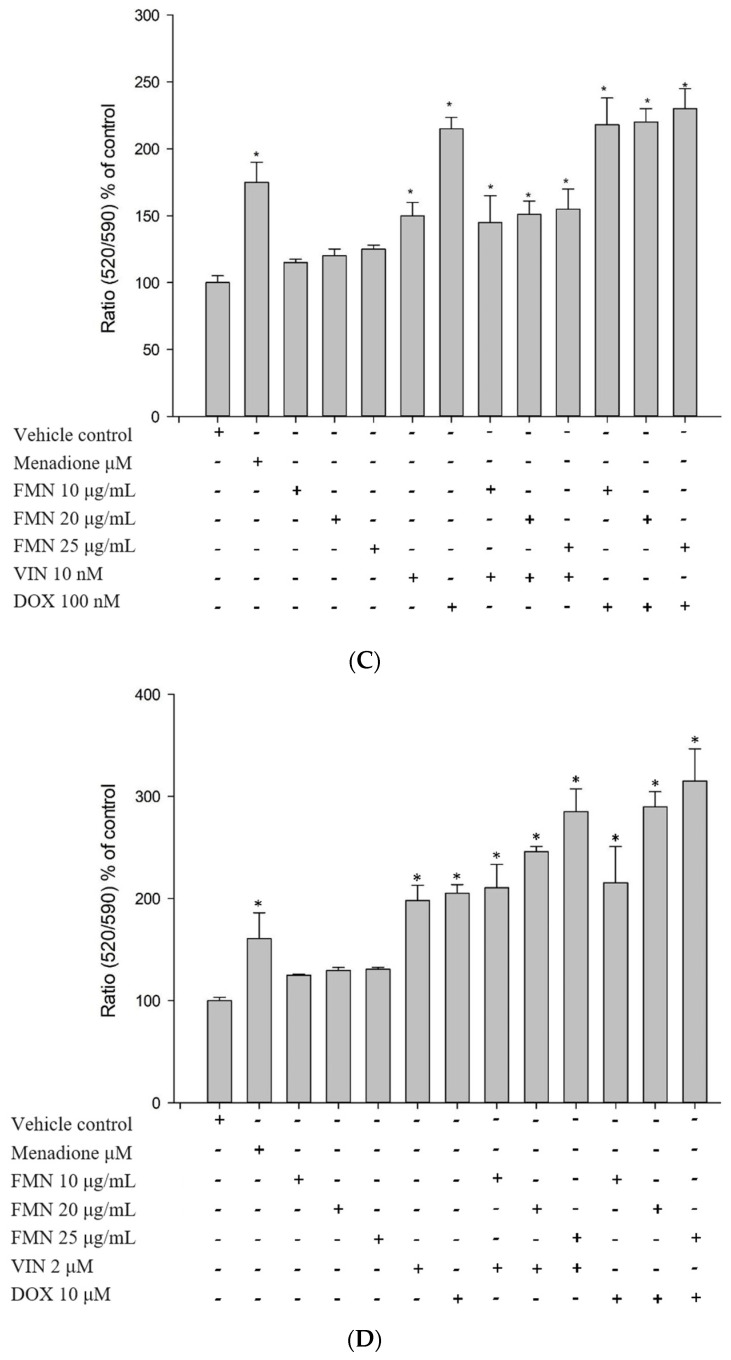

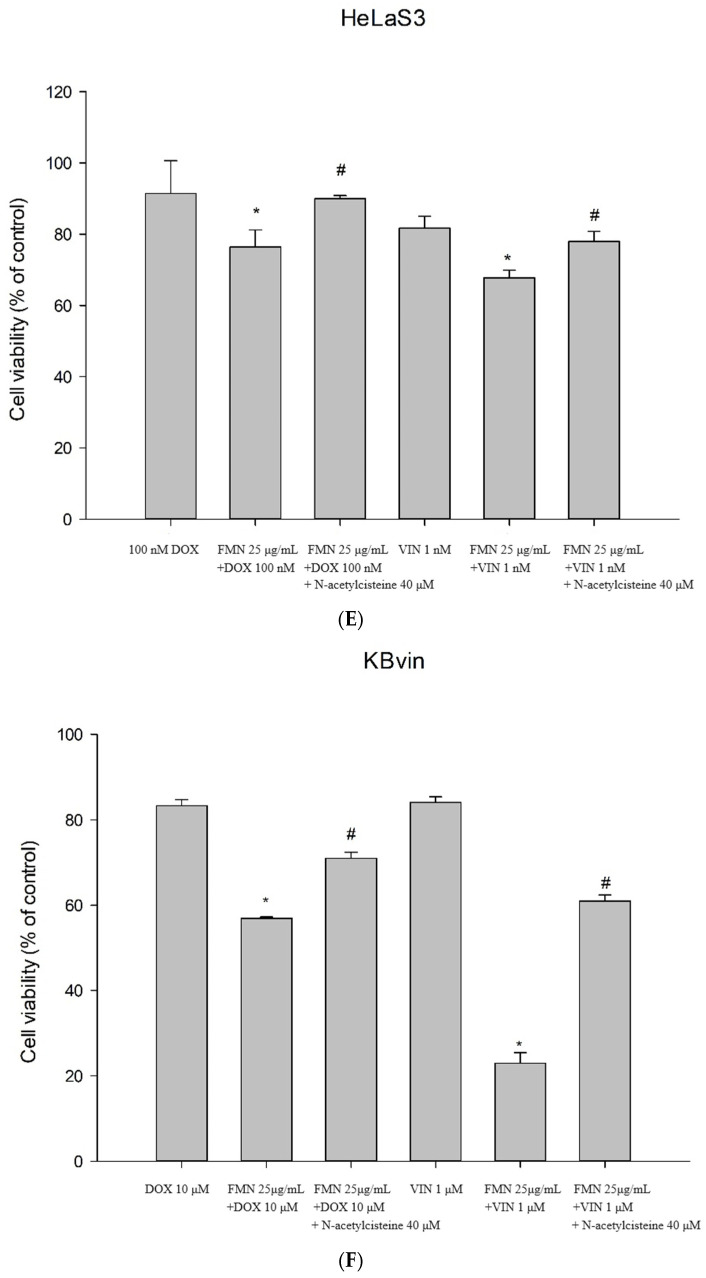
Intracellular ROS production and mitochondria membrane potential changes in the MDR KBvin cells. The intracellular ROS production was detected in the HeLaS3 (**A**) and KBvin cells (**B**). Formononetin with or without chemotherapeutic drugs were treated for 1 h. Menadione was used as a positive control. The mitochondria membrane potential changes were measured in the HeLaS3 (**C**) and KBvin cells (**D**). The cells were treated with formononetin or chemotherapeutic drugs only or formononetin combined with chemotherapeutic drug for 6 h. Menadione was used as a positive control. Each datum is expressed as the mean ± standard error of at least two experiments, each per-formed in triplicate. * denotes *p* < 0.05 as compared to control. N-acetylcysteine was applied as the antioxidant, and the cell viabilities were further evaluated by SRB assay in HeLaS3 (**E**) and KBvin cells (**F**). * denotes *p* < 0.05 as compared to with doxorubicin only or vincristine only group. # indicates *p* < 0.05 compared with formononetin combined doxorubicin or vincristine.

To testify whether the synergistic effect of formononetin was related to oxidative stress, intracellular ROS production and changes of mitochondria membrane potentials were examined. The intracellular ROS of HeLaS3 and MDR KBvin cells were significantly increased in the vincristine only, doxorubicin only, as well as in the combinations with formononetin (Figure 7A,B). The mitochondria membrane potential changes were also significantly increased in the vincristine only, doxorubicin only, as well as in the combinations with formononetin (Figure 7C,D). Combination treatment of N-acetylcysteine with formononetin significantly reduced the cytotoxicity of the co-treatment of doxorubicin or vincristine with formononetin in HeLaS3 cells (Figure 7E). Furthermore, the combination treatment of N-acetylcysteine with formononetin showed a significant influence on the formononetin MDR reversal effect in MDR KBvin cells (Figure 7F). These results demonstrated that N-acetylcysteine would partially eliminate the reversal effect of formononetin in MDR KBvin cells and indicated that the MDR reversal effect of formononetin may partially contribute to the enhanced oxidative stress.

### 2.7. Formononetin Resensitized MDR Cancer In Vivo

**Figure 8:**
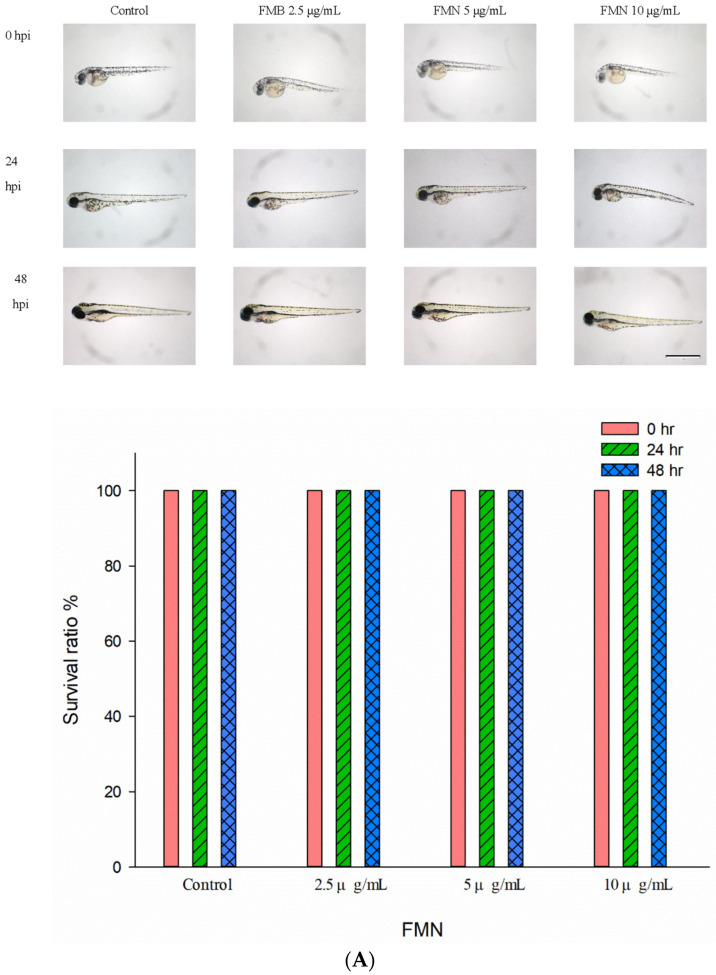

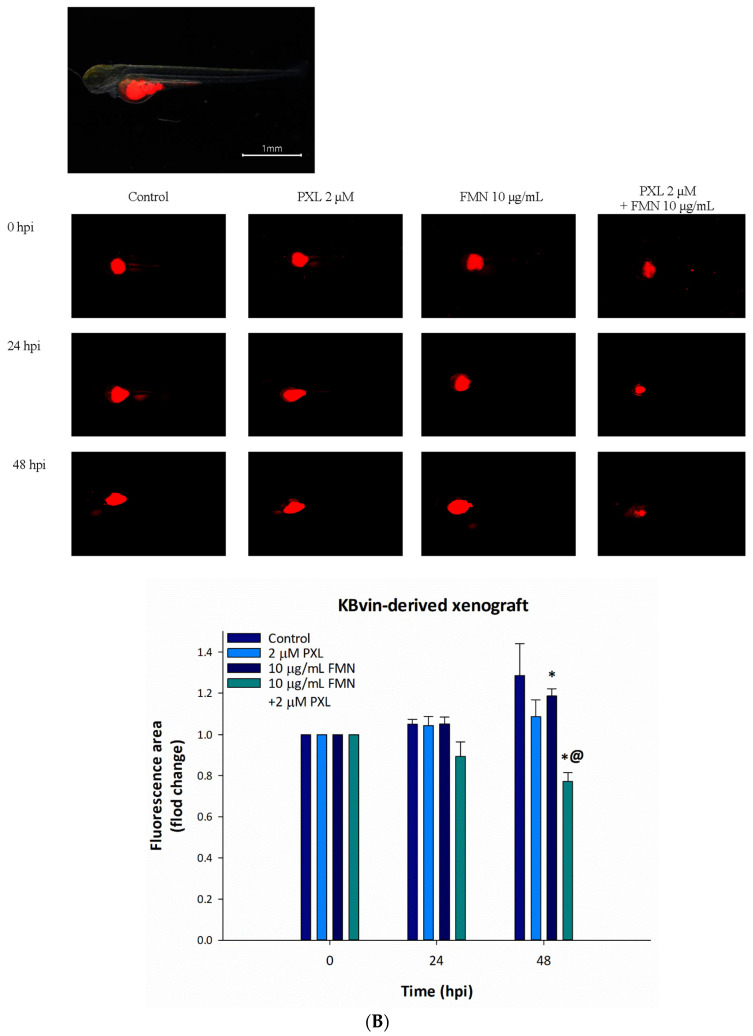
Formononetin combined with paclitaxel suppressed MDR KBvin cell growth in a xenotransplantation model. (**A**) Formononetin did not express significant toxicity at 48 hpi. (**B**) In an MDR KBvin xenograft model, we demonstrated that formononetin synergistically suppressed tumor size in combination with paclitaxel. The intensity of red fluorescence is proportional to the tumor size. Scale bar represents 1 mm. * *p* < 0.05 compared with the control; @ *p* < 0.05 compared to paclitaxel only group. hpf: hours post-fertilization; hpi: hours post-treatment or post-injection. The abbreviations of paclitaxel and formononetin are PXL and FMN, respectively.

A zebrafish xenograft model was performed to confirmed synergistically the suppression effect of formononetin on an MDR cancer cell in vivo. Figure 8A revealed that there was no toxicity observed with the indicated concentrations treatment. Figure 8B show that formononetin synergistically suppressed MDR cancer cell growth in vivo. 

## 3. Discussion

Chemotherapy remains the principal option for cancer treatment. Although the administration of chemotherapy has improved, many patients eventually experience MDR, resulting in cancer progress and relapse. The overexpression of P-gp is the most representative mechanism of MDR in numerous cancer types treated with chemotherapeutic agents and negatively correlated with chemosensitivity or survival [18]. Therefore, the targeted inhibitor P-gp is an efficacious solution for the treatment of MDR in human malignancies. Recently, the identification of novel or adjuvant anticancer agents derived from natural sources that have more curative effects and less toxicity has provided promising prospects for cancer treatments [10]. In this study, we found that formononetin, a natural flavonoid, considerably suppressed the P-gp transported ability through the ATPase stimulation and uncompetitively interrelated with P-gp transport of rhodamine 123 and doxorubicin. In addition, formononetin enhanced the chemosensitivity of MDR cancer cells at a non-cytotoxic concentration. Moreover, formononetin synergistically suppressed tumor growth in an MDR tumor xenograft model with paclitaxel. These results indicate that the synergistic effects of formononetin can be attributed to its P-gp inhibition ability rather than its cytotoxicity.

Formononetin is a naturally existing isoflavonoid, and previous studies demonstrated that the structural features of flavonoids effectively contribute to the inhibition of P-gp [10]. Of the natural flavonoids, quercetin displays P-gp modulatory and increased chemosensitivity abilities by inhibiting ATPase activity and the downregulation of *ABCB1* gene expression [19,20]. Taxifolin stimulated ATPase on P-gp, leading the inhibition of P-gp transported ability [21]. According to the effects on human P-gp ATPase activity, compounds are divided into three categories [22]. Most P-gp substrates are ATPase stimulators, except for high-affinity drugs such as tariquidar, which are reported to be P-gp ATPase inhibitors [23]. In the present study, formononetin accelerated basal and verapamil-stimulated P-gp ATPase activity, which is classified as a Class II agent.

The possible binding site on P-gp can be inferred by kinetic mechanism analysis. P-gp has two drug-binding sites, the H and R sites, and one modulator-binding site, the M site. Our analysis used rhodamine 123, which recognized the R and M sites, and doxorubicin, which only recognized the R site [24]. Our data reveal that formononetin interacts with rhodamine 123 and doxorubicin through uncompetitive inhibition. This suggests that formononetin-inhibited P-gp might not compete with rhodamine 123 or doxorubicin on the same drug-binding sites.

Formononetin has a wide range of anticancer effects, such as the induction of apoptosis and inhibition of cell differentiation, migration, invasion, and cell cycles in many cancer cell lines [14,25,26,27]. In one study, Liu et al. mentioned that FMN suppressed migration on nasopharyngeal cancer via the MAPK/ERK1/2 pathway [28]. Another study demonstrated that FMN exerted its effects, including chemotherapeutics cisplatin-induced hair cell death, through the PI3K/AKT-Nrf2 pathway [29]. Numerous flavonoids potentiated the efficacy of chemotherapies on several MDR cancer cell lines [7,30,31]. Two studies investigated the ability of formononetin to intensify chemosensitivity. One demonstrated that formononetin enhanced the cytotoxicity of epirubicin to human cervical cancer HeLa cells by inducing apoptosis and by suppressing the mRMA expression of MRP1 [32]. The other reported that 40 µg/mL formononetin increased the accumulation of rhodamine 123 in an MDR1-transfected mouse lymphoma cell line but did not enhance the antiproliferative activity of epirubicin in combination treatment. In this study, the MDR reversal potency of formononetin exhibited synergistic lethal and additive apoptosis effects when co-treated with chemotherapeutics.

In this study, we aimed to explore the P-gp inhibitory effects of formononetin on the transporter-specific expression system using stable cloned human P-gp expression cells (*ABCB1*/Flp-InTM-293) to avoid interference from other transporters. Additionally, the reversal ability of formononetin was evaluated using a drug-induced MDR cancer cell line to provide an authentic cancer environment in clinical MDR circumstances. Nonetheless, this study has some weaknesses. For example, the relatively high concentration of formononetin poses a challenge to its clinical application. However, formononetin could be considered a leading structure for the development of effective derivatives. Further in vivo investigations of its MDR reversal effect will be required.

## 4. Materials and Methods

### 4.1. Chemicals and Reagents

Formononetin was purchased from MedChemExpress^®^ (Monmouth Junction, NJ, USA). All the media and related reagents for cell culture were mentioned in a previous study [33]. All the chemical reagents, including calcein-AM, rhodamine 123, sulforhodamine B (SRB), trichloroacetic acid (TCA), and Tris Base were obtained from Sigma Chemical Co. (St. Louis, MO, USA).

### 4.2. Cell Lines and Cell Culture

The information on purchasing cells and generation of the MDR cell line have been mentioned in a previous study [33]. We used Flp-In^TM^-293 cell to generate P-gp overexpressing cells by transfecting with pcDNA5/*ABCB1* plasmid and pOG44 plasmid as previously described [34].

### 4.3. Rhodamine 123 and Doxorubicin Efflux Assay

The kinetics inhibition ability of formononetin was performed using P-gp standard fluorescent substrate rhodamine 123 and doxorubicin. We use these cells, Flp-In^TM^-293 cells and *ABCB1*/Flp-In^TM^-293 cells to detect the intracellular fluorescence of rhodamine 123 or doxorubicin efflux by P-gp as previously mentioned [34]. In terms of P-gp inhibition functional assays, such as calcein AM uptake assay, rhodamine 123 efflux assay and doxorubicin efflux assay, we applied the lowest effective P-gp inhibition concentration of formononetin. If we applied the same concentrations used in the MDR reversal assay, the fluorescence would exceed the highest detection limit of the instrument (BioTek Synergy HT Multi-Mode Microplate Reader, Winooski, VT, USA).

### 4.4. Determination of Cytotoxicity

The viability of all cell lines was measured using SRB assay, and the detailed information for experimental principles and procedures was declared in previous studies [35]. The brief protocol is mentioned below. A cell culture was placed into 96-well plates with each treatment for 3 days, and medium only was used as the control. Three days later, 50% TCA solution was added to fix the living cells, and 0.04% SRB was used for live cells staining. Using the 10 mM Tris base solution dissolved the staining cells. Finally, 515 nm absorbance was measured to represent the cell survival ratio. The synergy quantification of formononetin on MDR cells was calculated by CompuSyn 1.0 software.

### 4.5. Calcein–AM Uptake Assay

The theory of calcein–AM uptake assay for P-gp inhibition ability has been mentioned in a previous study [36]. Briefly, calcein–AM was a P-gp substrate which could be effluxed out of the cell by P-gp. Calcein that remained in the cell and underwent hydrolysis with fluorescence could be detected to represent the inhibiting ability of formononetin on P-gp efflux activity.

### 4.6. P-gp ATPase Assay

We used the recombinant human P-gp membranes, verapamil (positive control of drug-induced P-gp ATPase activity), Na_3_VO_4_ (the selective inhibitor of P-gp ATPase), and MgATP to measure the influence of formononetin on P-gp ATPase activity as outlined in a previous study [35].

### 4.7. MDR1 Shift Assay

The conformation-sensitive antibody of P-gp, UIC2, was used to identify whether formononetin was the substrate. The positive control and negative control of UIC2 binding assays were vinblastine and IgG2a antibody as previously mentioned [34].

### 4.8. Quantitative Real-Time RT-PCR

After exposure to formononetin for 72 h, the cells were collected and cellular RNA expression was quantitated by real-time RT-PCR. The mRNA expression of *ABCB1* was quantified by TaqMan Gene Expression Assay with Applied Biosystems StepOnePlus Real-Time PCR System (Waltham, MA, USA). GAPDH (Hs02758991_g1) was used as an internal control to normalize the level of human *ABCB1* (Hs00184500_m1) with the standard curve method. Detailed steps for the real-time reverse transcription polymerase chain reaction (RT-PCR) determination were mentioned in a previous study with minor modifications [36].

### 4.9. Apoptosis Assay

An additional apoptosis effect induced by formononetin was analyzed after 3 days of treatments with medium on, vincristine, and formononetin. All cells were collected and stained with 5 µL of Annexin V-FITC and propidium iodide (PI). Apoptotic cells were identified based on the manual (BD Pharmingen™ FITC Annexin V Apoptosis Detection Kit, San Diego, CA, USA).

### 4.10. Molecular Docking Simulations

The molecular docking procedure was performed by using the iGEMDOCK version 2.1. The structure of P-gp 3.6 A resolution (PDB ID: 6QEX) was obtained from the RCSB Protein Data Bank. During the docking analysis, the following parameters were applied: population size was 200 with generation 70, the number of solutions 2 with standard docking mode. The visualization and categorization of drug interactions and energy-based measurement function were provided by analyzing the results.

### 4.11. Measurement of Intracellular Total ROS Activity and Mitochondrion Membrane Potential Changes

The effect of formononetin on intracellular reactive oxygen species (ROS) was measured by a Cell Meter Fluorimetric Intracellular Total ROS Activity Assay Kit purchased from AAT Bioquest (Sunnyvale, CA, USA). The experimental procedures followed the manufactures’ protocol. Briefly, after cells were seeded for 24 h, the Amplite ROS Green working solution was added for 1 h. Formononetin with or without chemotherapeutic drugs was then added for 1 h at room temperature. The fluorescence was measured using a SpectraMax iD3 Multi-Mode Microplate Reader (Molecular Devices, LLC., San Jose, CA, USA) at 490/525 nm. To examine whether the reversal effect of formononetin comes from drug-induced oxidative stress, N-acetylcysteine was applied as the antioxidant, and the MDR reversal effect of formononetin was further evaluated by SRB assay.

The mitochondrial membrane potential change in formononetin was measured by JC-10 dye. Cells were cultured overnight and then treated with formononetin or chemotherapeutic drugs only or formononetin combined with chemotherapeutic drug for 6 h. The JC-10 dye working solution was dispensed to each well for 30 min at 37 °C. The fluorescence intensity was detected by a SpectraMax iD3 Multi-Mode Microplate Reader (Molecular Devices, LLC., San Jose, CA, USA).

### 4.12. Zebrafish Xenograft Assay

The in vivo effect of formononetin was demonstrated in a KBvin zebrafish xenograft model. Detailed information regarding the maintenance and animal experiments of zebrafish were mentioned as previously described [35]. All the tests were handled in conformity with the guidelines by the ethics committee of the Institutional Animal Care and Use Committee (IACUC) and the principles of 3Rs (Reduction, Replacement and Refinement) of China Medical University (China Medical University, Taichung, Taiwan). Each embryo was treated with formononetin or paclitaxel (as positive control) at the indicated concentrations for 24 and 48 h post-injection (hpi). Subsequently, the tumor size in each zebrafish was evaluated by inverted microscope (Nikon Eclipse TE2000-U, Tokyo, Japan).

### 4.13. Data and Statistical Analysis

The half-maximal inhibitory concentration, the IC_50_ value, was used to assess the inhibitory effect of test compounds and calculated as the following formula: E = E_0_ (IC50^s^/IC50^s^ + I^s^). E and E_0_ represented the inhibitory ability with and without compounds treatment. The concentration of inhibitors was denoted as I, and the half-maximal inhibitory concentration of test compounds was identified by IC_50_. S was the slope factor. Statistical differences in all data were measured using post hoc analysis (Tukey’s test) or Student’s *t*-test with ANOVA. If the *p*-value was less than 0.05, it indicated statistical significance.

## 5. Conclusions

This study demonstrated the finding of a natural product, formononetin, which is a potent inhibitor of human P-gp via uncompetitive inhibition and ATPase stimulation. Formononetin resensitized MDR cancer cells to chemotherapeutic drugs. The above results might provide new strategies for drug development in MDR cancer treatments.

## Figures and Tables

**Table 1:** The effects of formononetin on human P-gp-mediated efflux of rhodamine. 123 and doxorubicin in *ABCB1*/Flp-InTM-293 cells.

	Nonlinear Kinetic Parameters		
	V_m_ (pmol/mg protein/10 min)	K_m_ (μM)	
Nonlinear regression			
Rhodamine123 only	12.68 ± 0.67	19.28 ± 1.93	
+ formononetin 10 μM	6.11 ± 0.52 *	9.4 ± 1.11 *	
+ formononetin 20 μM	3.45 ± 0.1 *	4.81 ± 0.91 *	
efflux IC_50_ (μM)			9.04 ± 0.38
	V_m_ (pmol/mg protein/120 min)	K_m_ (μM)	
Nonlinear regression			
Doxorubicin only	55.99 ± 1.89	20.15 ± 2.83	
+ formononetin 10 μM	33.46 ± 0.95 *	11.9 ± 0.65 *	
+ formononetin 20 μM	23.6 ± 1.26 *	8.71 ± 2.51 *	
efflux IC_50_ (μM)			9.30 ± 0.14

V_m_, the maximal efflux rate; K_m_, the Michaelis–Menten constant. * *p* < 0.05 as compared with rhodamine 123 or doxorubicin only.

**Table 2:** Combination index analysis of vincristine and doxorubicin combined with formononetin at a non-constant ratio in MDR KBvin cells.

Chemotherapeutics Agent (nM)	Formononetin (µg/mL)	Fa *^a^*	CI *^b^*	Pharmacological Effect
Vincristine				
100	10	0.63	0.3	Strong Synergism
1000	10	0.22	0.1	Strong Synergism
100	20	0.60	0.5	Synergism
1000	20	0.25	0.2	Strong Synergism
100	25	0.51	0.5	Synergism
1000	25	0.20	0.2	Strong Synergism
Doxorubicin				
1000	25	0.65	0.7	Synergism
10,000	25	0.55	0.5	Synergism

*^a^* Fa: fraction affected; *^b^* CI: Combination Index.

## Data Availability

The data that support the findings of this study are available from the corresponding author upon reasonable request.
